# Supplementary material for: Differential requirements for Smarca5 expression during hematopoietic stem cell commitment
Source: Commun Biol. 2024 Feb 29;7:244. doi: 10.1038/s42003-024-05917-z (PMC10904812; doi:10.1038/s42003-024-05917-z)
Supplement: Supplementary file 2 — Supplementary Figs. 1-6 [file 42003_2024_5917_MOESM2_ESM.pdf]

a

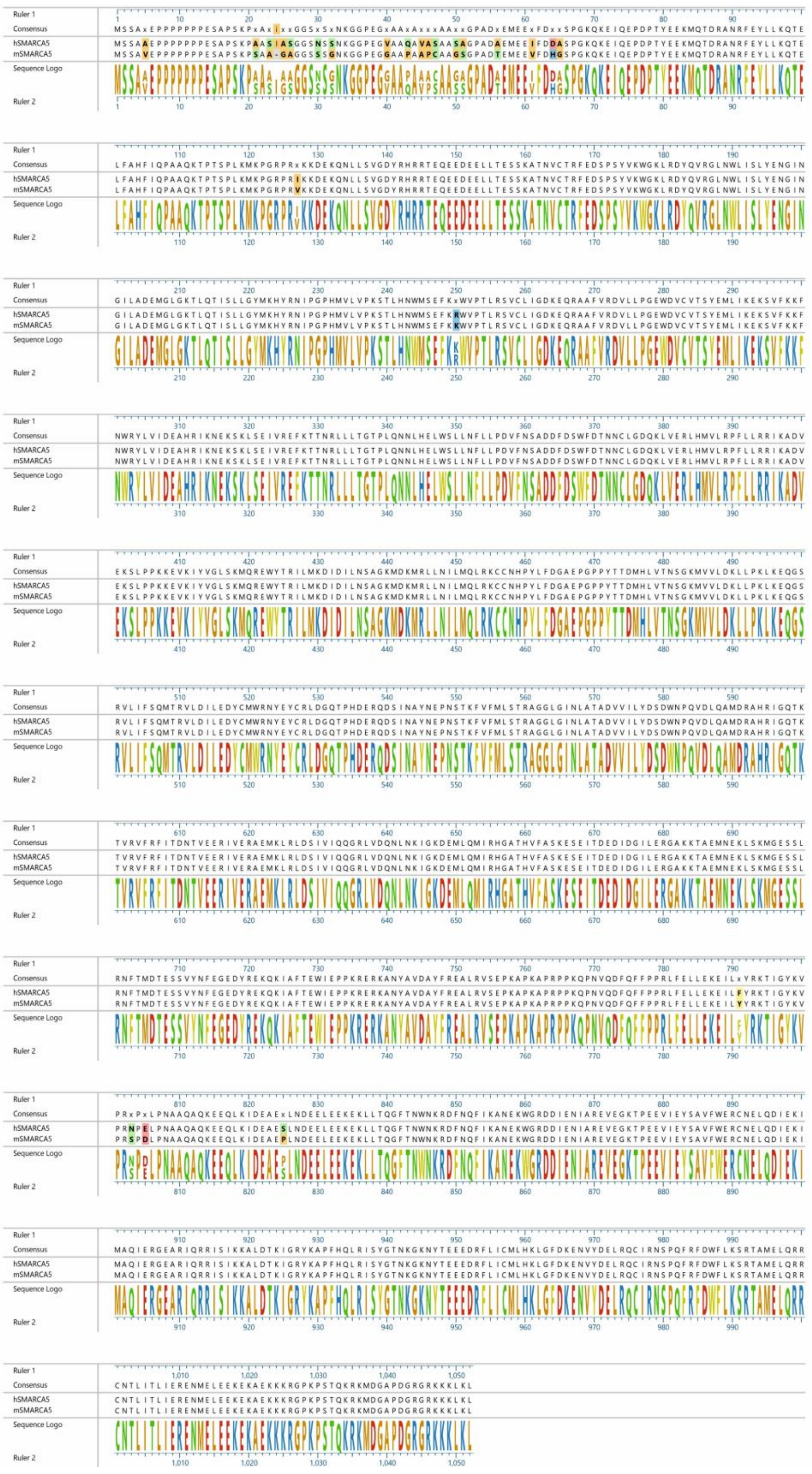

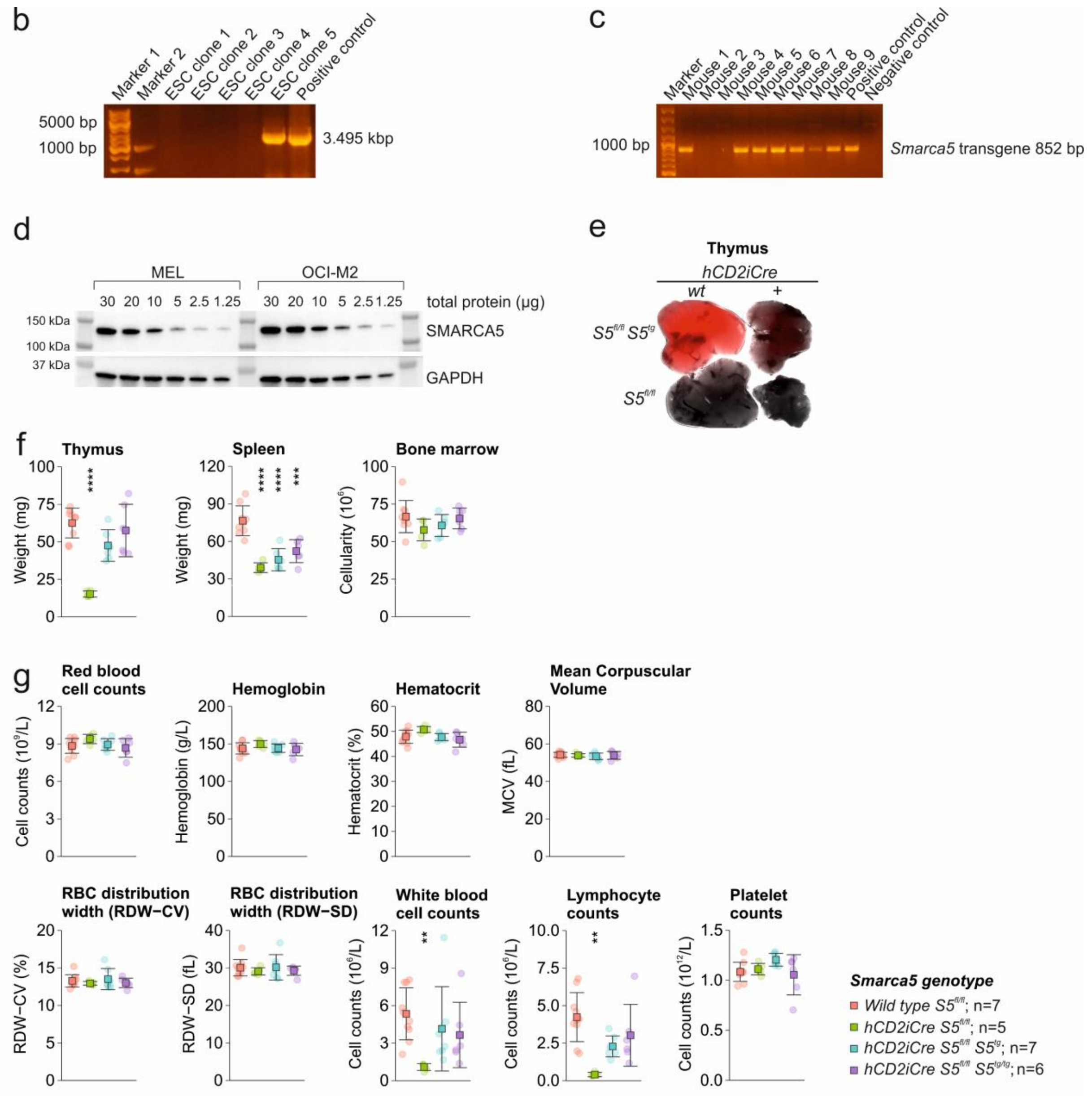

b
Marker 1
Marker 2
ESC clone 1
ESC clone 2
ESC clone 3
ESC clone 4
ESC clone 5
Positive control
5000 bp
1000 bp
3.495 kbp
c
Marker
Mouse 1
Mouse 2
Mouse 3
Mouse 4
Mouse 5
Mouse 6
Mouse 7
Mouse 8
Mouse 9
Positive control
Negative control
1000 bp
Smarca5 transgene 852 bp
d
MEL
OCI-M2
30 20 10 5 2.5 1.25
total protein (µg)
150 kDa
100 kDa
37 kDa
SMARCA5
GAPDH
e
Thymus
hCD2iCre
wt
+
S5fl/fl S5tg
S5fl/fl
f
Thymus
Spleen
Bone marrow
Weight (mg)
Cellularity (10⁶)
g
Red blood cell counts
Hemoglobin
Hematocrit
Mean Corpuscular Volume
Cell counts (10⁹/L)
Hemoglobin (g/L)
Hematocrit (%)
MCV (fL)
RBC distribution width (RDW−CV)
RBC distribution width (RDW−SD)
White blood cell counts
Lymphocyte counts
Platelet counts
RDW−CV (%)
RDW−SD (fL)
Cell counts (10⁶/L)
Cell counts (10¹²/L)
Smarca5 genotype
Wild type S5fl/fl; n=7
hCD2iCre S5fl/fl; n=5
hCD2iCre S5fl/fl S5tg; n=7
hCD2iCre S5fl/fl S5tg/tg; n=6

Supplementary Figure 1. Transgenic *SMARCA5* releases the blockade of T and B cell differentiation after *Smarca5* deletion. **(a)** Alignment of human and murine SMARCA5 amino acid sequence. **(b)** Electrophoretic gel (0.8 % agarose) with PCR analysis of mouse embryonic stem cells (ESC) with $S5^{tg}$ construct integrated in *Rosa26* locus. Positive band is 3.495 kbp long with fwd primer in *Rosa26* sequence and rev primer in *tdTomato* sequence. **(c)** Electrophoretic gel (1.5 % agarose) with PCR analysis of genomic DNA from chimeric mice with $S5^{tg}$ genotype. Positive band is 852bp long. **(d)** Western blot analysis of two leukemic cell lines MEL (murine erythroleukemia) and OCI-M2 (human adult acute myeloid leukemia) using antibody against SMARCA5. Numbers indicate total amount of protein loaded to the gel in micrograms. Staining with GAPDH were used as loading control. **(e)** Thymi of 8-week-old mice of indicated genotypes. Merge of visible light and fluorescence. The $S5^{tg}$ mouse (without Cre recombinase activation) expresses *tdTomato* (red), upon *hCD2iCre* activation, *tdTomato* is deleted and $S5^{tg}$ is expressed. **(f)** Thymus and spleen weight and bone marrow cellularity of indicated genotypes. Statistics: t-test relative to controls **(g)** Hematological measurements of peripheral blood from 2-month-old experimental animals of indicated genotypes. Statistics: One-Way ANOVA with Tukey's Honestly Significant Difference test (p adjusted value: p<0.05=*, p<0.01=**, p<0.001=***, p<0.0001=****, no asterisks = non-significant), the error bars represent standard deviation.

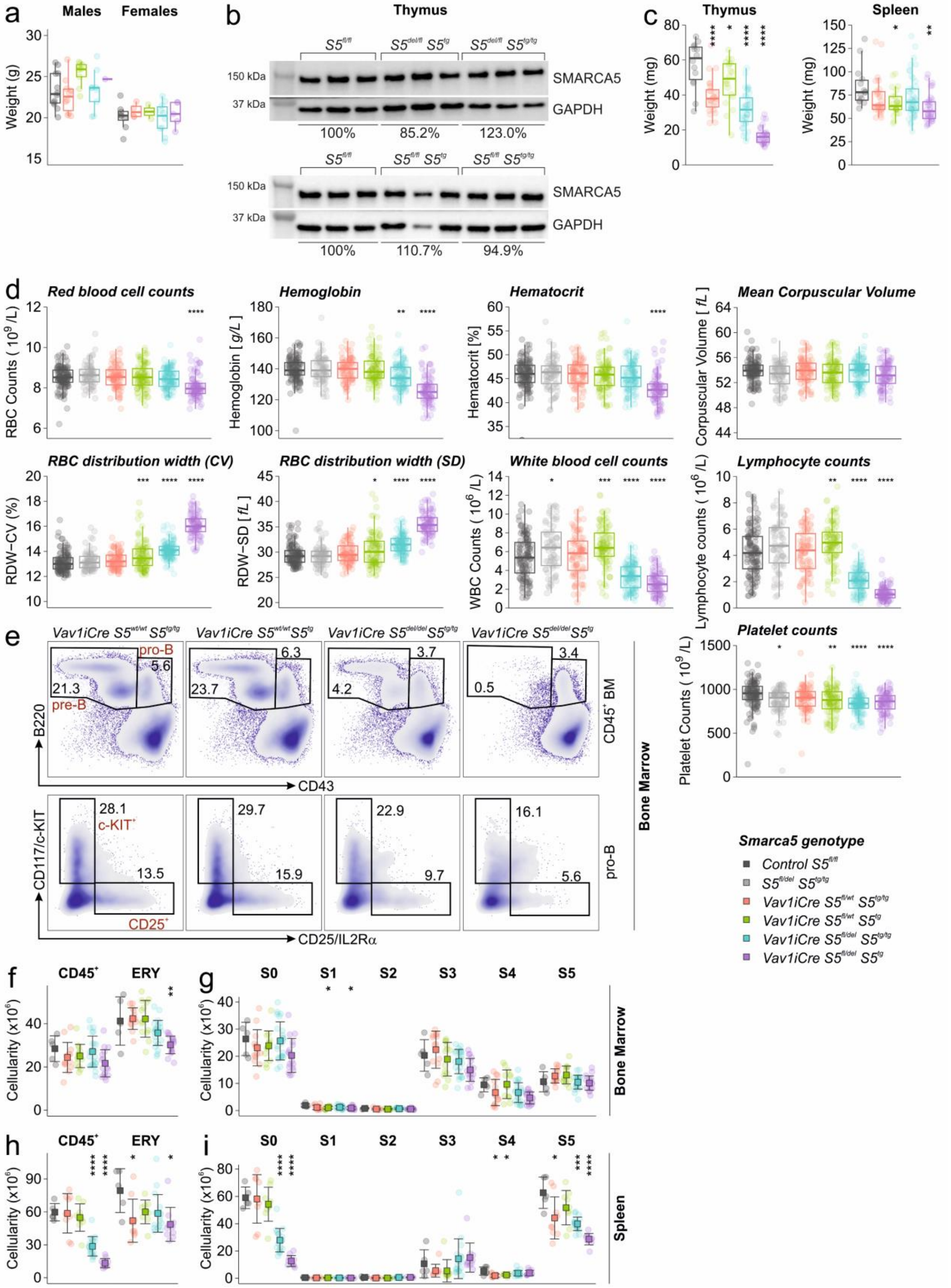
a
Males
Females
Weight (g)
b
Thymus
S5fl/fl
S5del/fl S5tg
S5del/fl S5tg/tg
150 kDa
37 kDa
SMARCA5
GAPDH
100%
85.2%
123.0%
S5fl/fl
S5fl/fl S5tg
S5fl/fl S5tg/tg
100%
110.7%
94.9%
c
Thymus
Spleen
Weight (mg)
d
Red blood cell counts
RBC Counts (10^9 /L)
Hemoglobin
Hemoglobin [g/L]
Hematocrit
Hematocrit [%]
Mean Corpuscular Volume
Corpuscular Volume [fL]
RBC distribution width (CV)
RDW−CV (%)
RBC distribution width (SD)
RDW−SD [fL]
White blood cell counts
WBC Counts (10^6 /L)
Lymphocyte counts
Lymphocyte counts (10^6 /L)
Platelet counts
Platelet Counts (10^9 /L)
e
Vav1iCre S5wt/wt S5tg/tg
Vav1iCre S5wt/wt S5tg
Vav1iCre S5del/del S5tg/tg
Vav1iCre S5del/del S5tg
pro-B
pre-B
21.3
5.6
23.7
6.3
4.2
3.7
0.5
3.4
B220
CD43
CD45+ BM
28.1
c-KIT+
13.5
CD25+
29.7
15.9
22.9
9.7
16.1
5.6
CD117/c-KIT
CD25/IL2Rα
pro-B
Bone Marrow
Smarca5 genotype
Control S5fl/fl
S5fl/del S5tg/tg
Vav1iCre S5fl/wt S5tg/tg
Vav1iCre S5fl/wt S5tg
Vav1iCre S5fl/del S5tg/tg
Vav1iCre S5fl/del S5tg
f
CD45+
ERY
Cellularity (x10^6)
g
S0
S1
S2
S3
S4
S5
Bone Marrow
h
CD45+
ERY
i
S0
S1
S2
S3
S4
S5
Spleen

Supplementary Figure 2. Mice expressing *SMARCA5* transgene in stem cells with simultaneous deletion of endogenous *Smarca5* have defects in T and B differentiation. (a) Weight of 2-month-old mice of the indicated genotypes and sex. **(b)** Immunoblot of thymi of 2-month-old mice of the indicated genotypes with anti-SMARCA5, antibody. For these experiments, we used mice with a non-conditional variant of the SMARCA5 transgene; the transgene was activated in the previous generation by ActB-Cre recombinase. As a result, the SMARCA5 transgene is non-conditionally expressed in every cell in the body of the analyzed mice. GAPDH staining was used as a loading control. Signal density is stated in % relative to controls. **(c)** Thymus and spleen weight of indicated genotypes. **(d)** Hematological measurements of peripheral blood from 2-month-old experimental animals of indicated genotypes. **(e)** Flow cytometry analysis of B-cell development in 2-month-old bone marrow (BM) of indicated genotypes. Upper panels: using B220 and CD43 staining of $CD45^+$ cells in BM. Lower panels: using CD117 and CD25 staining of pro-B cells ($CD43^+B220^+$). **(f, h)** Flow cytometry analysis of erythroid development in 2-month-old animals of indicated genotypes. Estimated size of $CD45^+$ and ERY ($Ter119^+$) populations in BM **(f)** and spleen **(h)**. **(g, i)** Estimated size of stage 0 to 5 (S0-S5) populations of erythroid development in 2-month-old animals distinguished using anti-Ter119 and anti-CD117/c-kit antibodies in BM **(g)** and spleen **(i)**. Statistics: One-Way ANOVA with Tukey's Honestly Significant Difference test (p adjusted value: p<0.05=*, p<0.01=**, p<0.001=***, p<0.0001=****, no asterisks = non-significant), the error bars represent standard deviation, for n numbers see Supplementary Table 1.

Supplementary Table 1.

Table of animals used in experiments in Figure 2 and Supplementary Figure 2:

	Ctrl $S5^{fl/fl}$	$S5^{fl/wt}$ $S5^{tg/tg}$	*Vav1iCre* $S5^{fl/wt}$ $S5^{tg/tg}$	*Vav1iCre* $S5^{fl/wt}$ $S5^{tg}$	*Vav1iCre* $S5^{fl/del}$ $S5^{tg/tg}$	*Vav1iCre* $S5^{fl/del}$ $S5^{tg}$
Fig. 2c, S2c, S2a Thymus	9		20	13	33	18
Fig. 2c, S2c Spleen	9		20	18	38	24
Fig. 2c, S2c Bone Marrow	9		27	26	47	31
Fig. 2d	45	13	21	22	30	25
Fig. 2e, 2f	9		10	2	21	6
Fig. 2g, 2h	9		9	13	23	16
Fig. S2d	117	68	78	96	107	84
Fig. S2f, S2g	9		13	13	23	17
Fig. S2h, S2i	9		9	9	17	10

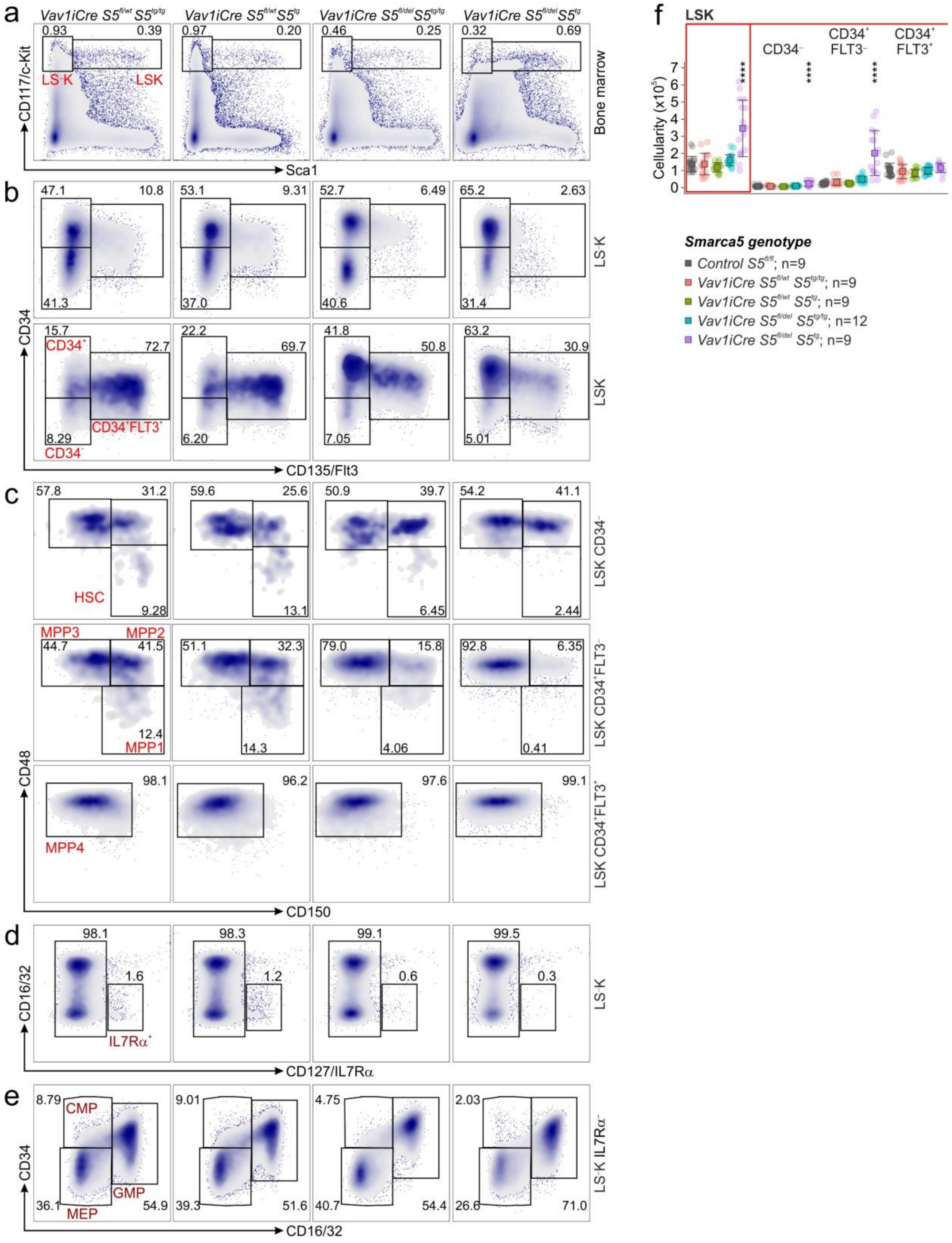
a
Vav1iCre S5fl/wt S5tg/tg
Vav1iCre S5fl/wt S5tg
Vav1iCre S5fl/del S5tg/tg
Vav1iCre S5fl/del S5tg
CD117/c-Kit
Sca1
LS-K
LSK
Bone marrow
b
CD34
CD135/Flt3
CD34+
CD34+FLT3+
CD34-
LS-K
LSK
c
HSC
MPP3
MPP2
MPP1
MPP4
CD48
CD150
LSK CD34-
LSK CD34+FLT3-
LSK CD34+FLT3+
d
CD16/32
CD127/IL7Rα
IL7Rα+
LS-K
e
CMP
GMP
MEP
CD34
CD16/32
LS-K IL7Rα-
f
LSK
CD34-
CD34+ FLT3-
CD34+ FLT3+
Cellularity (x10^5)
****
Smarca5 genotype
Control S5fl/fl; n=9
Vav1iCre S5fl/wt S5tg/tg; n=9
Vav1iCre S5fl/wt S5tg; n=9
Vav1iCre S5fl/del S5tg/tg; n=12
Vav1iCre S5fl/del S5tg; n=9

Supplementary Figure 3: Mice expressing *SMARCA5* transgene in stem cells with simultaneous deletion of endogenous *Smarca5* have impaired early hematopoietic development. (a) Flow cytometry of 10-week-old bone marrow from mice of the indicated genotypes. Analysis of c-Kit and Sca1 markers to exclude lineage antigens (CD3, Ly-6G/Ly-6C, CD11b, B220, Ter-119) yielded isolation of stem (LSK) and early progenitor ($LS^{-}K$) cells. **(b)** Flow cytometry of stem-cell marker CD34 and lymphoid/myeloid marker FLT3/CD135 in $LS^{-}K$ (upper panels) and LSK (lower panels) cells. **(c)** Cytometry of LSK $CD34^{-}$ (upper panels, long-term HSC) and LSK $CD34^{+}$ enriched multipotent progenitor (MPP) stem-cell populations (lower panels) differentiated by expression of SLAM markers CD48 and CD150. MPP1 – short-term HSCs, MPP2 – myeloid-oriented, MPP3 – lineage-balanced MPP. **(d)** Flow cytometry of lineage progenitor enriched $LS^{-}K$ cells in bone marrow using myeloid CD16/32 and lymphoid IL7Rα markers. **(e)** Flow cytometry of lineage-restricted ($CD127/Il7Ra^{-}$) progenitor subpopulations in LSK bone marrow cells using myeloid marker CD16/32 and CD34, MEP = megakaryocytic-erythroid progenitor, CMP = common myeloid progenitor, GMP = granulocyte-monocyte progenitor. **(f)** Absolute quantification of $CD34^{+/-}$ and $Flt3/CD135^{+/-}$ LSK cells. Statistics: One-Way ANOVA with Tukey's Honestly Significant Difference test (p adjusted value: p<0.05=*, p<0.01=**, p<0.001=***, p<0.0001=****, no asterisks = non-significant), the error bars represent standard deviation.

Supplementary Table 2.

Table of animals used in experiments in Figure 3i including total cell counts sorted from bone marrow of indicated genotypes:

Replicate number	Sex	Genotype	Sorted LSK	LSK count per replicate	Sorted LS-K	LS-K count per replicate
Rep1	F<br>M	*Ctrl* $S5^{fl/fl}$<br>*Ctrl* $S5^{wt/wt}$	87687<br>43149	130836	195736<br>194983	390719
Rep2	M<br>F	*Ctrl* $S5^{wt/wt}$<br>*Ctrl* $S5^{wt/wt}$	77272<br>65085	142357	261607<br>187996	449603
Rep3	F<br>M	*Ctrl* $S5^{fl/fl}$<br>*Ctrl* $S5^{wt/wt}$	43244<br>81017	124261	149213<br>219659	368872
Rep1	F<br>F	*Vav1iCre* $S5^{fl/fl}$ $S5^{tg/tg}$<br>*Vav1iCre* $S5^{fl/fl}$ $S5^{tg/tg}$	69210<br>92271	161481	131633<br>151216	282849
Rep2	F	*Vav1iCre* $S5^{fl/fl}$ $S5^{tg/tg}$	143015	143015	174271	174271
Rep3	M	*Vav1iCre* $S5^{fl/fl}$ $S5^{tg/tg}$	134538	134538	249369	249369
Rep1	F<br>M<br>F	*Vav1iCre* $S5^{fl/fl}$ $S5^{tg}$<br>*Vav1iCre* $S5^{fl/fl}$ $S5^{tg}$<br>*Vav1iCre* $S5^{fl/fl}$ $S5^{tg}$	79803<br>49073<br>52555	181431	71670<br>75996<br>26737	174403
Rep2	M<br>F	*Vav1iCre* $S5^{fl/fl}$ $S5^{tg}$<br>*Vav1iCre* $S5^{fl/fl}$ $S5^{tg}$	119203<br>56151	175354	123538<br>25823	149361
Rep3	F<br>M<br>F	*Vav1iCre* $S5^{fl/fl}$ $S5^{tg}$<br>*Vav1iCre* $S5^{fl/fl}$ $S5^{tg}$<br>*Vav1iCre* $S5^{fl/fl}$ $S5^{tg}$	76484<br>49324<br>43945	169753	85785<br>73652<br>27954	187391

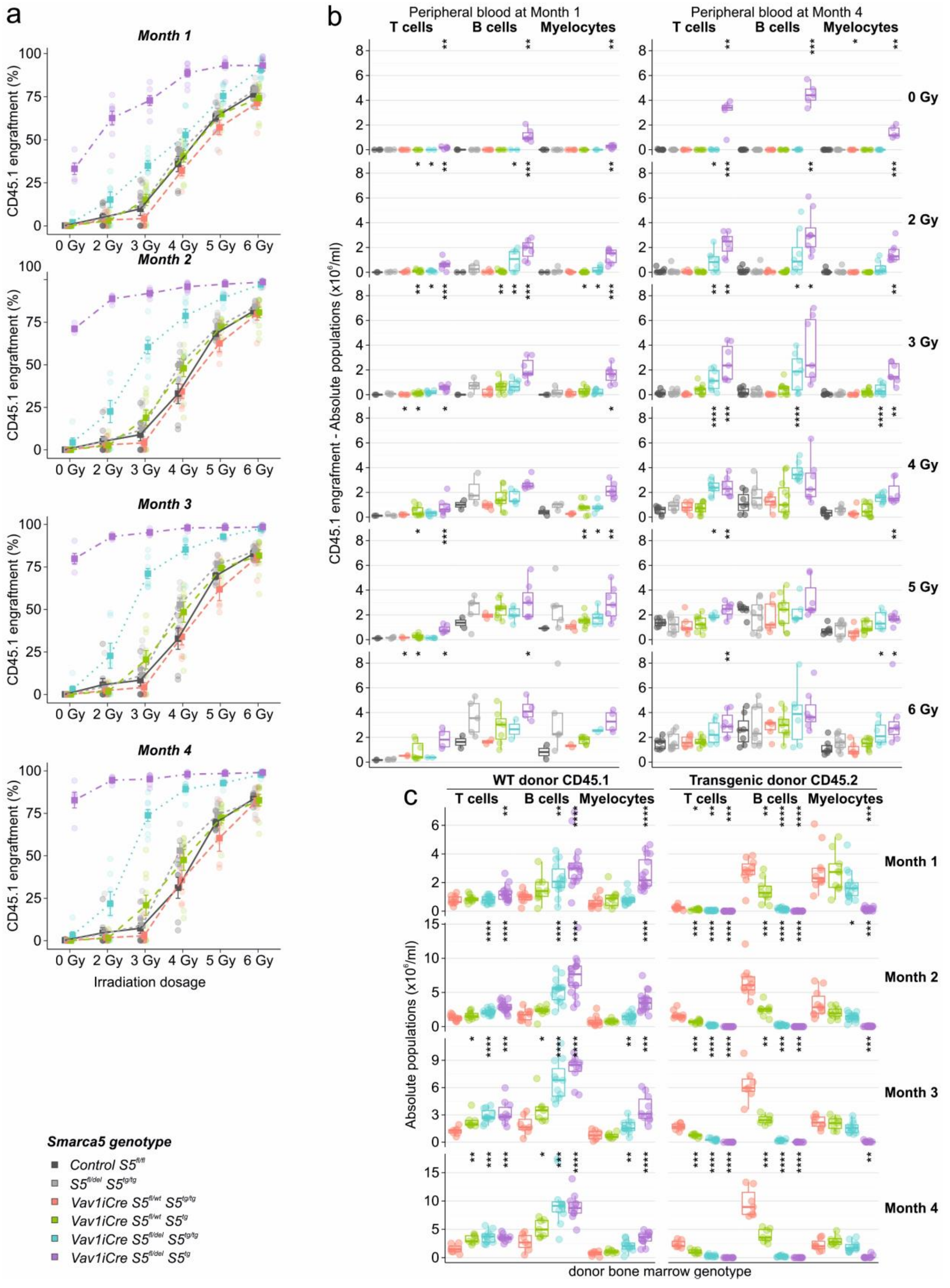
a
Month 1
Month 2
Month 3
Month 4
CD45.1 engraftment (%)
0 Gy 2 Gy 3 Gy 4 Gy 5 Gy 6 Gy
Irradiation dosage
Smarca5 genotype
Control S5^fl/fl
S5^fl/del S5^tg/tg
Vav1iCre S5^fl/wt S5^tg/tg
Vav1iCre S5^fl/wt S5^tg
Vav1iCre S5^fl/del S5^tg/tg
Vav1iCre S5^fl/del S5^tg
b
Peripheral blood at Month 1
Peripheral blood at Month 4
T cells B cells Myelocytes
0 Gy
2 Gy
3 Gy
4 Gy
5 Gy
6 Gy
CD45.1 engrafment - Absolute populations (x10^6/ml)
c
WT donor CD45.1
Transgenic donor CD45.2
T cells B cells Myelocytes
Month 1
Month 2
Month 3
Month 4
Absolute populations (x10^6/ml)
donor bone marrow genotype

Supplementary Figure 4: Transplantation of stem cells with only transgenic *SMARCA5* expression reveals defects in T and B cell repopulation. (a) Dose dependence of SMARCA5 expression levels (indicated genotypes) on graft repopulation (X-axis). Cytometry (%) of reconstitution of control CD45.1 donor bone marrow cells (BMCs) in $S5^{tg}$ recipient mice on CD45.2 background at 1 (short-term repopulation) to 4 months (long-term) after transplantation depending on irradiation dosage. **(b)** Absolute quantification (Y-axis) of engraftment of wild-type hematopoietic cells in bone marrow niches of the indicated genotypes. Quantification of hematopoietic lineage progeny (T, B, and myeloid cells, X-axis) after reconstitution of CD45.1 bone marrow (BM) donor cells from peripheral blood by flow cytometry at 1 to 4 months after transplantation depending on irradiation dosage. **(c)** Absolute quantification of competitive hematopoietic BM engraftment of the indicated genotypes. Peripheral blood progeny (T, B, and myeloid cells, X-axis) after reconstitution at 1 to 4 months. Statistics: t-test relative to controls (p<0.05=*, p<0.01=**, p<0.001=***, p<0.0001=****, no asterisks = non-significant), the error bars represent standard deviation, for n numbers see Supplementary Table 3.

Supplementary Table 3.

Table of animals used in transplantation experiments in Figure 4 and Supplementary Figure 4:

Fig. 4a, 4b, S4a, S4b	*Ctrl* $S5^{fl/fl}$	$S5^{fl/wt}$ $S5^{tg/tg}$	*Vav1iCre* $S5^{fl/wt}$ $S5^{tg/tg}$	*Vav1iCre* $S5^{fl/wt}$ $S5^{tg}$	*Vav1iCre* $S5^{fl/del}$ $S5^{tg/tg}$	*Vav1iCre* $S5^{fl/del}$ $S5^{tg}$
0 Gy	8	9	5	6	6	5
2 Gy	8	9	7	11	7	8
3 Gy	8	9	7	10	8	7
4 Gy	7	7	6	9	9	7
5 Gy	6	8	5	9	6	7
6 Gy	7	9	6	10	6	7

Fig. 4c, 4d, S4c	*Vav1iCre* $S5^{fl/wt}$ $S5^{tg/tg}$	*Vav1iCre* $S5^{fl/wt}$ $S5^{tg}$	*Vav1iCre* $S5^{fl/del}$ $S5^{tg/tg}$	*Vav1iCre* $S5^{fl/del}$ $S5^{tg}$
Month 1	10	9	14	17
Month 2	10	9	15	17
Month 3	10	9	15	17
Month 4	7	7	11	11

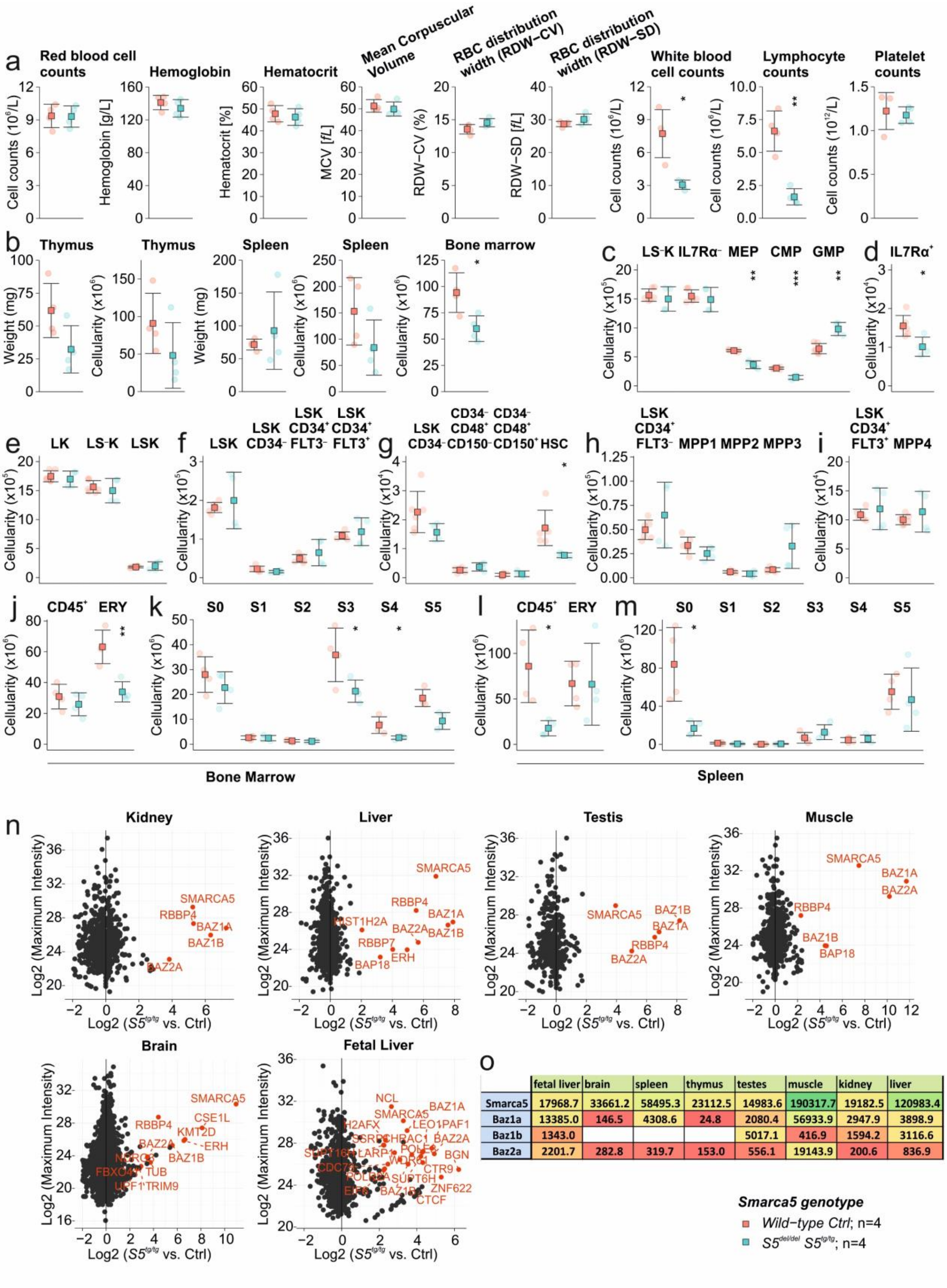

	fetal liver	brain	spleen	thymus	testes	muscle	kidney	liver
Smarca5	17968.7	33661.2	58495.3	23112.5	14983.6	190317.7	19182.5	120983.4
Baz1a	13385.0	146.5	4308.6	24.8	2080.4	56933.9	2947.9	3898.9
Baz1b	1343.0				5017.1	416.9	1594.2	3116.6
Baz2a	2201.7	282.8	319.7	153.0	556.1	19143.9	200.6	836.9

Supplementary Figure 5. The SMARCA5 transgenic product forms complexes and is essential for lymphopoiesis. (a) Hematological measurements of peripheral blood from 1-year-old experimental animals of indicated genotypes. **(b)** Thymus and spleen weight and thymus, spleen, and bone marrow cellularity of 1-year-old animals of indicated genotypes. **(c)** Flow cytometry analysis of the 1-year-old bone marrow of indicated genotypes. The expression of stem cell and progenitor markers c-Kit and Sca1 was used to identify bone marrow populations enriched for stem (LSK) and early progenitor cell populations ($LS^{-}K$). Lineage-positive (CD3, Ly-6G/Ly-6C, CD11b, B220, Ter-119) cells were excluded from all measurements. **(d)** Flow cytometry of stem cell marker CD34 and lymphoid/myeloid marker Flt3/CD135 in $LS^{-}K$ and LSK cells. **(e)** Flow cytometry of stem cell enriched LSK $CD34^{-}$ (HSC = long term-repopulating HSCs) and multipotent progenitor (MPP) enriched LSK $CD34^{+}$ populations distinguished by expression of SLAM markers CD48 and CD150. **(f)** Estimated sizes of $CD34^{+}$ LSK subpopulations enriched for hematopoietic progenitors with multi-lineage developmental potential (MPPs). MPP1 – short-term HSCs, MPP2 – myeloid-oriented, MPP3 – lineage-balanced MPP. **(g)** Estimated size of lymphoid-biased MPP4 population. **(h, i)** Quantification of lineage-restricted (CD127/$IL7R\alpha^{-}$) progenitor subpopulations **(h)** and early $IL7R\alpha^{+}$ lymphoid progenitors **(i)** in LSK bone marrow cells using myeloid marker CD16/32 and CD34. MEP = megakaryocytic-erythroid progenitor, CMP = common myeloid progenitor, GMP = granulocyte-monocyte progenitor. **(j, k)** Flow cytometry analysis of erythroid development in 1-year-old animals of indicated genotypes. Estimated size of $CD45^{+}$ and ERY ($Ter119^{+}$) populations in bone marrow **(j)** and spleen **(k)**. **(l, m)** Estimated size of stage 0 to 5 (S0-S5) populations of erythroid development in 1-year-old animals distinguished using anti-Ter119 and anti-CD117/c-kit antibodies in bone marrow **(l)** and spleen **(m)**. Statistics: t-test relative to controls ($p<0.05$=*, $p<0.01$=**, $p<0.001$=***, $p<0.0001$=****, no asterisks = non-significant), the error bars represent standard deviation. **(n)** Mass spectrometry (MS) analysis of SMARCA5 complexes in represented organs. Tissues from 2-month-old wild-type (negative control) and transgenic animals were lysed and tagged SMARCA5 was immunoprecipitated using FLAG-M2 agarose beads. **(o)** Table showing the intensity of MS data of SMARCA5 co-purified proteins from the depicted tissues. Only proteins with significantly higher intensities compared to control animals are shown in the table.

Fig. 1c

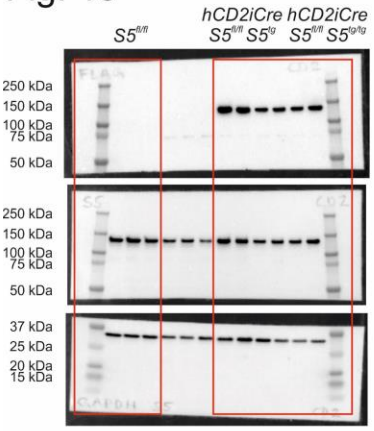

Suppl. Fig. 1c

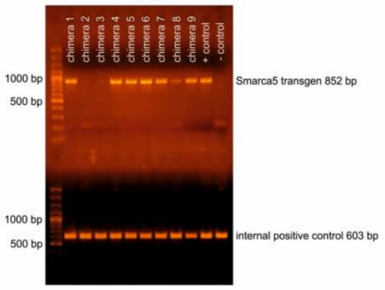

Suppl. Fig. 1d

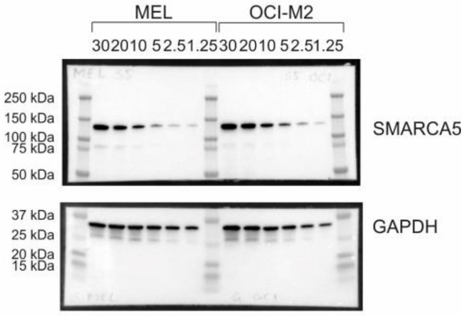

Fig. 2b

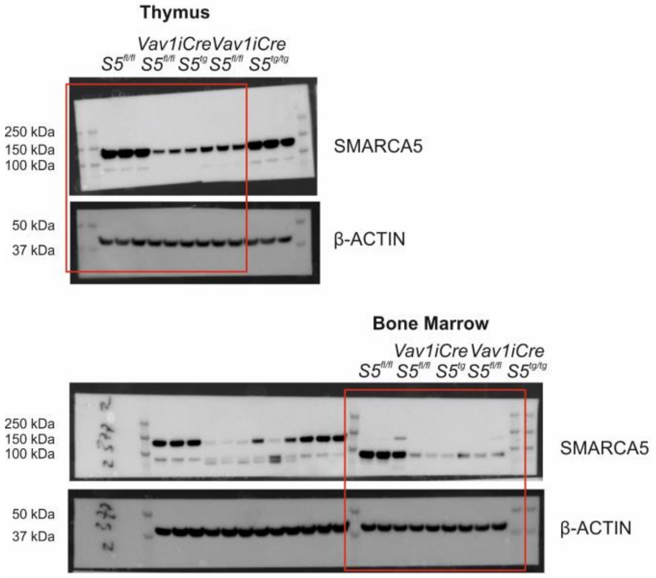

Suppl. Fig. 2b

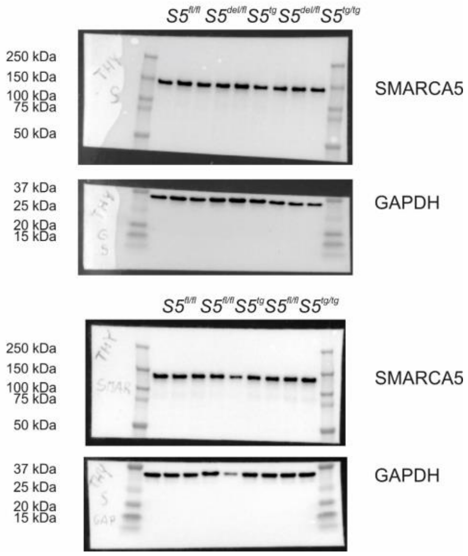

Supplementary Figure 6. Uncropped blots and gels.
