# Supplementary material for: Differential requirements for Smarca5 expression during hematopoietic stem cell commitment
Source: Commun Biol. 2024 Feb 29;7:244. doi: 10.1038/s42003-024-05917-z (PMC10904812; doi:10.1038/s42003-024-05917-z)
Supplement: Supplementary file 3 — Description of Additional Supplementary Files [file 42003_2024_5917_MOESM3_ESM.pdf]

Description of Additional Supplementary Files

File name: Supplementary Data

Description: A table of all source data used for the graphs shown in the figures, with the exception of the proteomics data that was uploaded to the online databases, see the Data Availability Statement. The figures are divided into different sheets according to the type of experiment (RT-qPCR, FACS, etc.) and also according to different tissues (thymus, bone marrow, etc.). Specific genotypes are listed in the tables.
