# Supplementary material for: Differential requirements for Smarca5 expression during hematopoietic stem cell commitment
Source: Commun Biol. 2024 Feb 29;7:244. doi: 10.1038/s42003-024-05917-z (PMC10904812; doi:10.1038/s42003-024-05917-z)
Supplement: Supplementary file 5 — Reporting Summary [file 42003_2024_5917_MOESM5_ESM.pdf]

nature portfolio

Corresponding author(s): Professor Tomas Stopka
tstopka@lf1.cuni.cz
Dr Lukas Cermak
lukas.cermak@img.cas.cz

Last updated by author(s): Nov 15, 2023

Reporting Summary

Nature Portfolio wishes to improve the reproducibility of the work that we publish. This form provides structure for consistency and transparency in reporting. For further information on Nature Portfolio policies, see our Editorial Policies and the Editorial Policy Checklist.

Please do not complete any field with "not applicable" or n/a. Refer to the help text for what text to use if an item is not relevant to your study.
For final submission: please carefully check your responses for accuracy; you will not be able to make changes later.

Statistics

For all statistical analyses, confirm that the following items are present in the figure legend, table legend, main text, or Methods section.

n/a	Confirmed	
☐	☒	The exact sample size (*n*) for each experimental group/condition, given as a discrete number and unit of measurement
☐	☒	A statement on whether measurements were taken from distinct samples or whether the same sample was measured repeatedly
☐	☒	The statistical test(s) used AND whether they are one- or two-sided<br>*Only common tests should be described solely by name; describe more complex techniques in the Methods section.*
☐	☒	A description of all covariates tested
☐	☒	A description of any assumptions or corrections, such as tests of normality and adjustment for multiple comparisons
☐	☒	A full description of the statistical parameters including central tendency (e.g. means) or other basic estimates (e.g. regression coefficient) AND variation (e.g. standard deviation) or associated estimates of uncertainty (e.g. confidence intervals)
☒	☐	For null hypothesis testing, the test statistic (e.g. *F*, *t*, *r*) with confidence intervals, effect sizes, degrees of freedom and *P* value noted<br>*Give P values as exact values whenever suitable.*
☒	☐	For Bayesian analysis, information on the choice of priors and Markov chain Monte Carlo settings
☒	☐	For hierarchical and complex designs, identification of the appropriate level for tests and full reporting of outcomes
☒	☐	Estimates of effect sizes (e.g. Cohen's *d*, Pearson's *r*), indicating how they were calculated

Our web collection on statistics for biologists contains articles on many of the points above.

Software and code

Policy information about availability of computer code

Data collection	No custom data collection software was used.
Data analysis	Data was analyzed using openly available R/Bioconductor packages.

For manuscripts utilizing custom algorithms or software that are central to the research but not yet described in published literature, software must be made available to editors and reviewers. We strongly encourage code deposition in a community repository (e.g. GitHub). See the Nature Portfolio guidelines for submitting code & software for further information.

Data

Policy information about availability of data

All manuscripts must include a data availability statement. This statement should provide the following information, where applicable:

- Accession codes, unique identifiers, or web links for publicly available datasets
- A description of any restrictions on data availability
- For clinical datasets or third party data, please ensure that the statement adheres to our policy

All data supporting the findings of this study are available within the manuscript and its supplementary information.

Research involving human participants, their data, or biological material

Policy information about studies with human participants or human data. See also policy information about sex, gender (identity/presentation), and sexual orientation and race, ethnicity and racism.

Reporting on sex and gender	No human research participants were involved.
Reporting on race, ethnicity, or other socially relevant groupings	-
Population characteristics	-
Recruitment	-
Ethics oversight	-

Note that full information on the approval of the study protocol must also be provided in the manuscript.

Field-specific reporting

Please select the one below that is the best fit for your research. If you are not sure, read the appropriate sections before making your selection.

☒ Life sciences ☐ Behavioural & social sciences ☐ Ecological, evolutionary & environmental sciences

For a reference copy of the document with all sections, see nature.com/documents/nr-reporting-summary-flat.pdf

Life sciences study design

All studies must disclose on these points even when the disclosure is negative.

Sample size	No sample size calculation was performed. Each experiment consisted of at least 3 biological replicates (exact n numbers are given for each experiment). For flow cytometry data at least 1000 events are reported for rarest cell types.
Data exclusions	No data was excluded from experiment, except low quality samples/measurements.
Replication	Multiple flow cytometry, mass spectrometry, western blot and RT-qPCR measurements were performed using cells from all described tissues and genotypes to ensure reproducibility of the experimental findings. Transplantation experiments were performent using multiple mice - exact n nubers are provided in supplementary materials. All experiments were reproducible.
Randomization	Littermates and samples from age matched mice were used always when available.
Blinding	No blinding was used. All data experimental data was collected upon sample availability.

Reporting for specific materials, systems and methods

We require information from authors about some types of materials, experimental systems and methods used in many studies. Here, indicate whether each material, system or method listed is relevant to your study. If you are not sure if a list item applies to your research, read the appropriate section before selecting a response.

Materials & experimental systems

n/a	Involved in the study	
☐	☒	Antibodies
☒	☐	Eukaryotic cell lines
☒	☐	Palaeontology and archaeology
☐	☒	Animals and other organisms
☒	☐	Clinical data
☒	☐	Dual use research of concern
☒	☐	Plants

Methods

n/a	Involved in the study	
☒	☐	ChIP-seq
☐	☒	Flow cytometry
☒	☐	MRI-based neuroimaging

Antibodies

Antibodies used	Antibodies for flow cytometry, all antibodies supplied by BioLegend (San Diego, California, USA) with exception for SV-AF700: Biotin anti-B220 (clone RA3-6B2; cat. no. 103204), FITC anti-B220 (clone RA3-6B2; cat. no. 103206), APC anti-CD4 (clone GK1.5; cat. no.

100412), BV421 anti-CD4 (clone GK1.5; cat. no. 100437), PE/Cy7 anti-CD5 (clone 53-7.3; cat. no. 100621), APC anti-CD8a (clone 53-6.7; cat. no. 100712), Biotin anti-CD11b/Mac-1 (clone M1/70; cat. no. 101204), BV421 anti-CD11b/Mac-1 (clone M1/70; cat. no. 101236), Biotin anti-CD11c (clone N418; cat. no. 117304), BV510 anti-CD16/32 (clone 93; cat. no. 101333), BV605 anti-CD25 (clone PC61; cat. no. 102036), PE anti-CD25 (clone PC61; cat. no. 102008), Biotin anti-CD34 (clone HM34; cat. no. 128604), APC anti-CD43 (clone S11; cat. no. 143208), FITC anti-CD44 (clone IM7; cat. no. 103006), PE/Cy7 anti-CD45 (clone 30-F11; cat. no. 103114), AF700 anti-CD45.1 (clone A20; cat. no. 110724), PE/Cy7 anti-CD45.2 (clone 104; cat. no. 109830), FITC anti-CD48 (clone HM48-1; cat. no. 103404), PE anti-CD71 (clone RI7217; cat. no. 113808), BV421 anti-CD117/c-Kit (clone 2B8; cat. no. 105828), BV786 anti-CD127/IL7Ra (clone A7R34; cat. no. 135037), APC anti-CD135/FLT3 (clone A2F10; cat. no. 135310), BV605 anti-CD150 (clone TC15-12F12.2; cat. no. 115927), Biotin anti-Gr-1 (clone RB6-8C5; cat. no. 108404), BV605 anti-Gr-1 (clone RB6-8C5; cat. no. 108440), Biotin anti-Nk1.1 (clone PK136; cat. no. 108704), PE anti-Nk1.1 (clone PK136; cat. no. 108708), FITC anti-Sca1 (clone D7; cat. no. 108106), PE anti-Sca1 (clone D7; cat. no. 108108), APC anti-Ter119 (clone Ter-119; cat. no. 116212), Biotin anti-Ter119 (clone Ter-119; cat. no. 116204), PerCP anti-Ter119 (clone Ter-119; cat. no. 116226), AF700 anti-mouse Lineage Cocktail with Isotype Ctrl (anti-CD3, clone 17A2; anti-Ly-6G/Ly-6C, clone RB6-8C5; anti-CD11b, clone M1/70; anti-CD45R/B220, clone RA3-6B2; anti-TER-119/Erythroid cells, clone Ter-119; cat. no. 133313), SV-PE/Cy7 (cat. no. 405206), SV-AF700 (cat. no. S21383; Life Technologies Corporation, Eugene, Oregon, USA).
Primary antibodies for immunoblotting: anti-SMARCA5 (1:1,000; Bethyl Laboratories, Montgomery, TX, USA; cat. no. A301-017A; Lot: 1), anti-FLAG (1:1,000; Cell Signaling, Danvers, MA, USA; cat. no. 2368; Lot: 12), anti-β-ACTIN (1:1,000; Abcam, Cambridge, UK; cat. no. ab6276) and anti-GAPDH (1:1,000; Sigma-Aldrich, Saint Louis, MO, USA; cat. no. HPA040067; Lot: 000015810).
Secondary antibodies for immunoblotting: anti-Rabbit IgG secondary antibody (1:10,000; Jackson ImmunoResearch, Cambridgeshire, UK; cat. no. 711-036-152), anti-Mouse IgG secondary antibody (1:10,000; Jackson ImmunoResearch, Cambridgeshire, UK; cat. no. 715-036-150).

Validation

Flow cytometry - Single color or fluorescence minus one (FMO) controls were used to validate antibody specificity.
Immunoblotting - Antibodies were validated experimentally using negative and positive controls where applicable.

Animals and other research organisms

Policy information about studies involving animals; ARRIVE guidelines recommended for reporting animal research, and Sex and Gender in Research

Laboratory animals

Mouse C57Bl/6NCrl, both sexes, age is specified in figure legends.

Wild animals

Study did not involve wild animals.

Reporting on sex

Animals of both sexes were used for this study, we did not distinguish between sexes in our analysis as we did not see any differences between males and females.

Field-collected samples

Study did not involve field-collected samples.

Ethics oversight

All experiments met the criteria approved by Czech Ministry of Agriculture and the Committee for Experimental Animals.

Note that full information on the approval of the study protocol must also be provided in the manuscript.

Flow Cytometry

Plots

Confirm that:

☒ The axis labels state the marker and fluorochrome used (e.g. CD4-FITC).

☒ The axis scales are clearly visible. Include numbers along axes only for bottom left plot of group (a 'group' is an analysis of identical markers).

☒ All plots are contour plots with outliers or pseudocolor plots.

☒ A numerical value for number of cells or percentage (with statistics) is provided.

Methodology

Sample preparation

A single cell suspensions from thymus and spleen were obtained using Dounce homogenizer. Samples from peripheral blood were incubated at RT and single cell suspensions from bone marrow, thymus, and spleen were incubated on ice with specific primary antibodies in PBS + 1 % biotin-free BSA solution for 20 minutes. Biotinylated primary antibodies were then visualized using streptavidin-conjugated fluorescent dyes.

Instrument

BD LSRFortessaTM SORP (BD Biosciences, San Jose, CA, USA) or CytoFLEX (Beckman Coulter, Brea, CA, USA)

Software

FlowJo FC analysis software

Cell population abundance

Cell fraction abundance was determined by antibody staining. For flow cytometry data at least 1000 events are reported for rarest cell types.

Gating strategy

Experiments use standard gating strategy, FSC-A/FSC-H, SCC-A/SCC-H and FSA-A/SCA-A for eliminations of dublets and cell debris, when possible cell were stained by viability dye (propidium iodide). Most of gating strategies are provided in

manuscript and supplemenary figures (LSK, MPP, B cell development). Gating strategy for thymocytes (CD4/CD8 and DN1-4) was similar as in (Zikmund, Kokavec et al. 2019). Further gating strategy information can be provided if needed.

☐ Tick this box to confirm that a figure exemplifying the gating strategy is provided in the Supplementary Information.

This checklist template is licensed under a Creative Commons Attribution 4.0 International License, which permits use, sharing, adaptation, distribution and reproduction in any medium or format, as long as you give appropriate credit to the original author(s) and the source, provide a link to the Creative Commons license, and indicate if changes were made. The images or other third party material in this article are included in the article's Creative Commons license, unless indicated otherwise in a credit line to the material. If material is not included in the article's Creative Commons license and your intended use is not permitted by statutory regulation or exceeds the permitted use, you will need to obtain permission directly from the copyright holder. To view a copy of this license, visit http://creativecommons.org/licenses/by/4.0/
